# Temporal Processing Evaluation in Tinnitus Patients: Results on Analysis of Gap in Noise and Duration Pattern Test

**Published:** 2013-10

**Authors:** Vahid Mehdizade Gilani, Masumeh Ruzbahani, Parvane Mahdi, Amin Amali, Mohammad Hossein Nilforush Khoshk, Jalal Sameni, Alireza Karimi Yazdi, Hamed Emami

**Affiliations:** 1 *Department of Audiology, Faculty of Rehabilitation. Tehran University of Medical Sciences, Tehran, Iran.*; 2 *Department of Otorhinolaryngology-Head and Neck Surgery, Imam Khomeini Educational Complex Hospital, Tehran University of Medical Sciences, Tehran, Iran.*; 3 *Department of Audiology, Faculty of Rehabilitation. Isfahan University of Medical Sciences, Isfahan, Iran.*

**Keywords:** Duration pattern test, Gap in noise test, Temporal processing, Tinnitus

## Abstract

**Introduction:**

Tinnitus is a perception of sound without external source. For complete assessment of tinnitus, central auditory processing abilities should be considered in addition to the routine psychological evaluation of tinnitus characteristics. Temporal processing is one of the important auditory skills that are necessary for complex higher level auditory processing.

**Materials and Methods:**

20 tinnitus patients and 20 healthy volunteers without tinnitus, all with normal auditory thresholds (≤ 20 dBnHL), were enrolled in present study. Pure Tone Audiometry (PTA), Tinnitus evaluation, Gap in Noise (GIN) test and Duration Pattern Test (DPT) were applied to all participants.

**Result:**

Analysis of GIN test revealed statistically significant increases in an approximate threshold value of gap detection in the patients group, both in right and left sides (P=0.007 and P=0.011, respectively). Comparison of percentage of correct responses in between two groups was also statistically meaningful in right and left ears (P=0.019 and P=0.026, respectively). The comparison of different parameters of DPT in two study groups revealed no significant differences in percentage of correct responses between two groups (P>0.05).

**Conclusion:**

GIN test results identified auditory temporal resolution difficulties in patients with tinnitus, meaning that in spite of normal auditory thresholds there may be some possibility of abnormality in central auditory processing functions.

## Introduction

Tinnitus by definition is a phantom perception of sound without external source (1,2). It can occur at any age as well as in 1–3% of the general population. Tinnitus is associated with a variety of disorders in the auditory system, whether generated peripherally or centrally, but may arise spontaneously, too (3,4). Evaluation of tinnitus sufferers may not be complete without assessing the effect of tinnitus on different auditory skills. 

Temporal processing is defined as an ability to process acoustic stimuli over the time. This is of significant concern given the fact that this basic auditory behavior is very crucial, at least in part, for most complex higher level processing, such as speech perception in quiet and in background noise, localization, discrimination, pattern processing, binaural integration, and binaural separation (5,6). 

As there is evidence that patients with tinnitus experience difficulty in understanding degraded speech, the evaluation of temporal processing in these patients are self-evidenced. The results of such a study provide important information regarding the relationship between peripheral or central mechanisms of tinnitus generation that may affect auditory processing.

Several recent studies reported application of the acoustic startle reflex in order to measure gap detection in rats to assess temporal processing in these animals. The results indicated that rats exhibiting noise induced tinnitus had deficiency in detecting silent gaps when compared to the controls and suggested that tinnitus may affect temporal processing (7,8). 

Actually, gap detection impairments have been considered in animals as a tool to objectify the presence of tinnitus (8). With regards to literature in temporal processing of humans with tinnitus, the purpose of the present study was to provide a quantitative assessment in this field by applying Gap In Noise (GIN) and Duration Pattern Test (DPT). As these tests are easy to apply and have a high sensitivity and specificity as well as evaluate each ear separately, they are two commonly used measures of temporal processing. It was hypothesized that neural activity in tinnitus patients might create deficits in their ability in temporal processing when compared to those non-tinnitus individuals.

## Materials and Methods

A cross sectional study was performed on 20 patients (10 female and 10 male) suffering from tinnitus but with normal hearing thresholds, attending the otorhinolaryngoly clinic in a Imam Khomeyni Educational Complex Hospital, from June 2010 to August 2011, and on 20 healthy volunteers (10 female and 10 male) without tinnitus, as a control group. The inclusion criteria for the patient group were applied at baseline history of a tinnitus complaint for at least one year duration, age of more than 18 years and less than 45 years, normal hearing status (threshold of ≤ 20 dBHL) and tympanogram.

Following obtaining informed consent and local ethics committee approval all partici- pants underwent the test procedures as follow.

*Pure tone Audiometry:*

Following otoscopic examination, partici- pants underwent audiometric test using a an Amplaid 311 diagnostic audiometer(Amplaid, Italy) at the frequencies of 0.25,0.5,1.0,2.0,4.0 and 8.0 KHz for air conduction and between 0.25 and 4.0 KHz for bone conduction. 

The psychoacoustic characteristics of the tinnitus were evaluated in all patients in order to define the pitch and loudness of the perceived tinnitus. Pitch matching attempts to quantify tinnitus in terms of its possible frequency, in that, two tones were presented to the patient and the patients were asked to choose which one most closely matches the tinnitus that they hear. In loudness matching the perceptual equivalent of sound intensity was defined in term of decibel, and the procedure started at a level just below threshold and increased intensity until the patient signaled a match (8,9).

*Gap in Noise (GIN) test:*

One of the tests to assess the temporal resolution in the present study was the GIN test, developed by Musiek et al, in 2005 (6). A compact disk with the recordings of the stimuli was played in a CD player coupled to the calibrated audiometer. The test materials consisted of series of 6s white noise stimuli, in which 0 to 3 silence gaps of different durations (2-6,8,10,12,15,20 ms) were embedded within each segment. The stimuli were presented at 50 dB SL with regard to the speech recognition threshold (SRT) to each ear, separately. The task required was to identify the silence gaps, in milliseconds (ms), distributed along a white noise presentation. The parameters of interest were approximate gap detection threshold, i.e., the shortest silence gap noticed by the subject at least four out of six times and the percentage of correct responses out of total gaps. 

*Duration Pattern Test (DPT):*

This is the minimum difference in duration necessary to perceive that two otherwise identical stimuli are different in duration. It consisted of a sequence of three consecutive 1000 Hz tones with one differing by being Longer (L), 500 msec, or Shorter (S), 200 msec, in duration than the other two tones in the sequence. Six different sequences (LLS, LSL, LSS, SLS, SLL and SSL) were used in the test. The presentation level was 50 dBSL with regard to the patient’s SRT. The subjects were instructed to respond to each stimulus presentation with a verbal description of the sequence heard. The parameters of interest were the percentage of correct responses.

*Statistical analysis:*

SPSS software, version 11.5 (Chicago, IL, USA), was used for statistical evaluation. 

The normalcy of data distribution was evaluated by means of the Kolmogorov-Smirnov test. Differences in mean values of parameters between patient and control group were analyzed by student t test. Furthermore, correlations between different parameters were assessed using Pearson’s correlation. In all statistical procedures, instances with a P value less than 0.05 were considered to be statistically significant.

## Results

In the sample of the consecutively admitted 20 tinnitus patients, the mean age was 30.31 ± 9.35 years (range,19–45 years) and for the control subjects it was 27.80±7.74 years (range, 18–45 years). There was no statistically significant difference between these two groups according to gender and age (P>0.05). The median duration of the tinnitus complaint in patients was 7 years (range, 2-13 years). In patients group, 14 (70.00%) had bilateral and 6 (30.00%) had unilateral tinnitus, with the laterality that two had right ear and four had left ear involvement. 

Table 1 compares the GIN test findings from the patient group (with tinnitus) and the control (without tinnitus) group. Statistically significant increases were noticed in an approximate threshold value of gap detection in patients group, both in right and left sides (P=0.007, P= 0.011, respectively), indicating that tinnitus patients needed a longer duration of gap to detect than those of the non-tinnitus subjects. Comparison of percentage of correct responses in between two groups was also statistically meaningful in right and left ears (P=0.019, P=0.026, respectively). At the same time, considering different genders, disparities of GIN parameters did not reach a statistically significant level in either of the two groups (P>0.05); evidencing that gender did not influence the test results (Table. 1). 

**Table 1:** GIN Test Results

	**Threshold/ Right**	**Threshold/ Left**	**Percentage of Correct Response/ Right**	**Percentage of Correct Response/ Left**
Control Subjects	4.95±0.21	4.80±0.11	71±1.22	70±1.31
Patients Subjects	6.15±0.32	6.15±0.28	64±2.22	61±1.78
P- Value	0.011	0.026	0.007	0.019

Table.2 illustrates the comparison of different parameters of DPT in the two study groups. There was neither a meaningful difference in percentage of correct responses between two study groups nor between two genders in each group (P>0.05) (Table. 2).

**Table 2:** DPT Results

	**Percentage of Correct Response/ Right**	**Percentage of Correct Response/ Left**	
Control Subjects	96.00±1.00	98.00±0.60	
Patients Subjects	97.00±0.87 98.00±0.54		
P- Value	0.219		0.106

## Discussion

Tinnitus is an otologic symptom and despite the great amount of research in this subject, the exact pathophysiological process of tinnitus remains unclear. Involvement of the whole auditory system, either peripheral or central, should be considered in the development of tinnitus. Some authors postulated that the presence of tinnitus has been associated to a disorder in the neural activity of the auditory system. A cochlear disorder, even when undiagnosed by pure tone audiometry, may initiate a series of processes in the nervous system that may result in tinnitus (1,9). Ami et al (2008) and Hesse et al (2005), carried out Distortion Product Oto Acoustic Emission (DPOAE) in tinnitus sufferers and revealed that reduced outer hair cell activity, as detected by reduced DPOAE levels, may manifest as tinnitus even prior there is a shift on hearing threshold (10,11).

Additionally, according to some published data, deficits in neural structures in central auditory nervous system may result in the perception of tinnitus (3,12,13). Bartels et al (2007), stated that an altered afferent input to the auditory pathway may be the initiator of a complex sequence of events, conclusively resulting in the generation of tinnitus at the central level of the auditory nervous system (13). 

Assessment of central auditory processing in different group of patients is one of the audiologist’s scopes of practice. Musiek et al (2005), performed GIN in subjects with confirmed central auditory nervous system involvement and reported a statistically significant increase in gap detection thresholds (6), indicating that the GIN test holds promise as a clinically useful tool in the assessment of temporal resolution, one of the central auditory abilities, in the clinical arena. Sepehrnejad et al (2011) compared GIN test results between congenitally blind and sighted subjects with normal hearing and described that there was a significant difference in the approximate threshold and the percent of corrected answers between two groups suggesting of compensative auditory neuroplasticity after visual deprivation (14). Sanches et al (2010) applied GIN test to assess the auditory temporal resolution skill in 18 tinnitus patients and 23 normal participants and reported that control group detected gaps with a shorter time interval than the patients group (15). Haas et al (2012) also pointed out threshold values of gap detection in tinnitus patients were longer in duration than non-tinnitus subjects and hypothesized that some changes in neural activity in tinnitus patients might prolong Gap Detection Threshold (GDT) (16). Fournier and Hebert (2012), assessed gap detection in human with tinnitus and postulated that tinnitus group displayed a consistent deficit in gap processing at both low and high background noise frequencies, assuming that ongoing tinnitus masks the gap and results in their impaired gap detection (8). In the present study, the higher approximate threshold and the fewer correct responses showed that the tinnitus patients need longer duration of gap in order to correct gap detection, and in accordance to the literature data, revealed poorer temporal acuity abilities. 

From physiological view points, detection of a silence gap embedded in the noise, requisites the fine processing of temporal structures of sound and is dependent on the integral auditory system for perfect transmission of acoustic information through auditory pathway (6,17). Werner et al (2001) stated that mechanisms underlying gap detection are not well understood. To some extent gap detection depends mostly on processes within, or peripheral to, the auditory brainstem (18).

However, analysis of DPT did not reveal any significant differences in between the two study groups. Correct perception of duration pattern requires the normal function of right and left hemisphere (5). Musiek et al (1990) demonstrated that in split-brain patients there was an abnormality in DPT results. They reported the sensitivity and specificity of this test 86% and 92%, respectively, for cerebral deficits, however DPT was so resistant to the lower level pathologies such as cochlear involvement and was affected by neither hearing loss nor the small loss of system resolution (5). Consequently, the normal result of DPT in tinnitus patients was due to insensitivity of this test to the abnormalities of structures below the auditory cortex.

## Conclusion

In the present study, the GIN test results identified auditory temporal resolution difficulties in patients with tinnitus, meaning that in spite of normal auditory thresholds there may be some potential abnormality in central auditory processing functions of these patients. Further studies using other techniques in order to evaluation of auditory processing are desired in order to more precise diagnosis for better remediation.
